# Heterogeneous muscle gene expression patterns in patients with massive rotator cuff tears

**DOI:** 10.1371/journal.pone.0190439

**Published:** 2018-01-02

**Authors:** Michael C. Gibbons, Kathleen M. Fisch, Rajeswari Pichika, Timothy Cheng, Adam J. Engler, Simon Schenk, John G. Lane, Anshu Singh, Samuel R. Ward

**Affiliations:** 1 Department of Bioengineering, University of California San Diego, La Jolla, California, United States of America; 2 Department of Computational Biology, University of California San Diego, La Jolla, California, United States of America; 3 Department of Orthopedic Surgery, University of California San Diego, La Jolla, California, United States of America; 4 Department of Orthopedic Surgery, Kaiser Permanente, San Diego, La Jolla, California, United States of America; 5 Department of Radiology, University of California San Diego, La Jolla, California, United States of America; Mayo Clinic Minnesota, UNITED STATES

## Abstract

Detrimental changes in the composition and function of rotator cuff (RC) muscles are hallmarks of RC disease progression. Previous studies have demonstrated both atrophic and degenerative muscle loss in advanced RC disease. However, the relationship between gene expression and RC muscle pathology remains poorly defined, in large part due to a lack of studies correlating gene expression to tissue composition. Therefore, the purpose of this study was to determine how tissue composition relates to gene expression in muscle biopsies from patients undergoing reverse shoulder arthroplasty (RSA). Gene expression related to myogenesis, atrophy and cell death, adipogenesis and metabolism, inflammation, and fibrosis was measured in 40 RC muscle biopsies, including 31 biopsies from reverse shoulder arthroplasty (RSA) cases that had available histology data and 9 control biopsies from patients with intact RC tendons. After normalization to reference genes, linear regression was used to identify relationships between gene expression and tissue composition. Hierarchical clustering and principal component analysis (PCA) identified unique clusters, and fold-change analysis was used to determine significant differences in expression between clusters. We found that gene expression profiles were largely dependent on muscle presence, with muscle fraction being the only histological parameter that was significantly correlated to gene expression by linear regression. Similarly, samples with histologically-confirmed muscle distinctly segregated from samples without muscle. However, two sub-groups within the muscle-containing RSA biopsies suggest distinct phases of disease, with one group expressing markers of both atrophy and regeneration, and another group not significantly different from either control biopsies or biopsies lacking muscle. In conclusion, this study provides context for the interpretation of gene expression in heterogeneous and degenerating muscle, and provides further evidence for distinct stages of RC disease in humans.

## Introduction

The progressive and irreversible loss of rotator cuff (RC) muscle that occurs in RC disease is a vexing clinical challenge[1]. Despite advances in surgical tools and techniques, outcomes of RC repair are often unsatisfactory, especially for those with large tendon tears and chronic, advanced disease[1]. These suboptimal outcomes include tendon re-tear and persistent functional limitations[2], and occur in a significant number of cases[3]. Compositional changes on a macroscopic scale can in part explain these outcomes, as muscle volume is displaced by fat[4]. Changes at the tissue level may also be responsible for poor outcomes, as muscle fiber organization and force production are reduced with tear[5], and patients with the most severe RC disease (those undergoing RSA) demonstrate widespread muscle fiber degeneration[6]. To better understand the biological processes that govern muscle loss and fatty infiltration in RC disease, several studies have evaluated gene expression in human RC muscle[7, 8]. Here, we aim to address a key limitation of previous studies by correlating gene expression to histological biopsy composition, and provide potential interpretations of our findings as they relate to progression of RC disease.

Two previous studies of human RC muscle gene expression showed that when compared to small tears, large or massive tears generally exhibit depressed expression of key myogenic, adipogenic, and fibrotic genes along with high myostatin expression[7, 8], suggestive of an anti-myogenic disease process[9, 10]. However, a major limitation of these and many other molecular studies of heterogeneous tissues is the difficulty of reconciling gene expression values with changes in tissue composition, which could influence measured transcript abundance[11]. Given the gross changes in muscle composition observed across the spectrum of RC diseases[6, 12, 13], it is reasonable to hypothesize that gene expression changes are driven as much by the composition (e.g. muscle content) of the tissue as by changes in gene expression that occur within a given tissue type or cell population, a measurement which itself remains difficult[14]. Despite this common assumption, that gene expression patterns are influenced by changes in the underlying tissue composition, no previous study has included both gene expression and compositional data. Therefore, two major aims of this work were to 1) generate evidence to determine whether and to what extent tissue composition predicts gene expression patterns, and 2) use those findings to provide context for and caution against interpretation of gene expression data in the absence of compositional data.

Beyond the technical limitations of previous studies, we placed an emphasis on patients with advanced RC disease in this study, as these patients typically have the most severe muscle loss and the poorest outcomes among patients with cuff tears. We were particularly interested in genes and pathways involved in muscle atrophy and regeneration along with adipogenic and fibrotic genes, given the apparent irreversibility of muscle loss and fat and fibrotic tissue accumulation following chronic, massive RC tear. By providing insight into the relationship between gene expression and tissue composition, we hope to provide some perspective and context for previous studies while offering insight into the biological processes that govern the latter stages of RC disease.

## Materials and methods

### Patients

**Fig 1:**
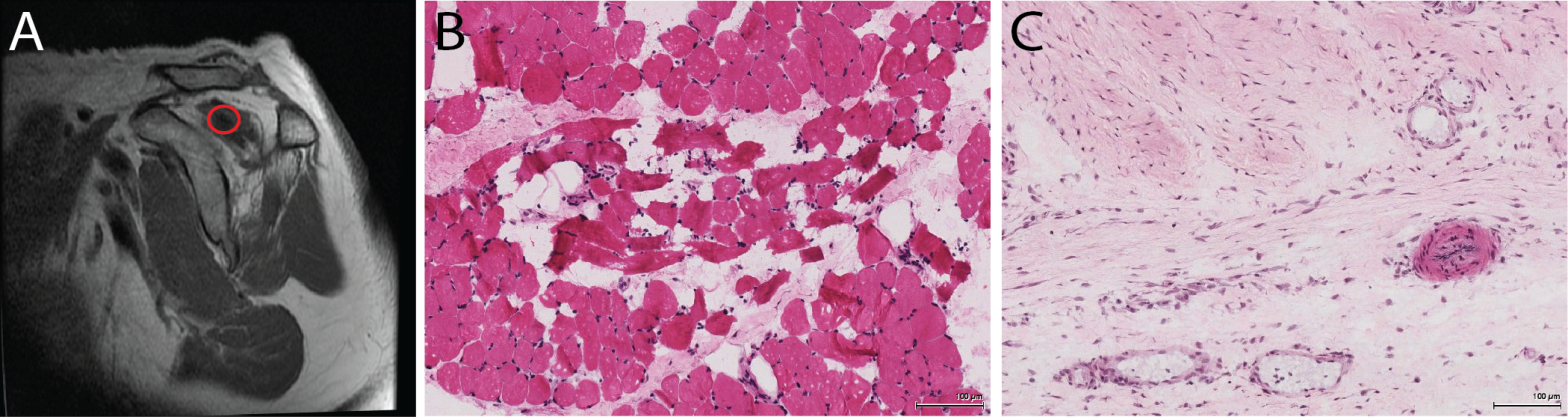
(A) MRI demonstrating the approximate biopsy region, where only regions of apparent muscle were targeted. (B) Representative H&E image of a muscle-containing biopsy, with high levels of muscle degeneration. (C) Representative H&E image of a biopsy that did not contain muscle, demonstrating high cellularity and presence of larger vascular structures.

Twenty-three patients undergoing reverse total shoulder arthroplasty (RSA) were consented for RC muscle biopsy. All biopsies were performed with informed written consent under the approval of the UC San Diego IRB (study #090829). In order to specifically target muscle, obvious regions of tendon and fat were avoided and only samples that macroscopically appeared vascular and organized in fascicular structures were retained (red circle in Fig 1A). The supraspinatus was always biopsied if tissue meeting these criteria was present, otherwise the infraspinatus or teres minor were biopsied using the same criteria. In some cases both supraspinatus and infraspinatus were biopsied in order to increase the number of samples containing myofibers, leading to a total of 31 RSA biopsies, for which the histological data has been published[6]. Samples from patients with intact RC tendon were obtained arthroscopically from the superficial and lateral surface of the supraspinatus muscle during subacromial decompression surgeries (n = 9). After biopsy, samples were flash frozen in liquid nitrogen-cooled isopentane and stored at -80°C for future processing.

### RNA isolation and quantitative PCR

For gene expression analysis, biopsy cross-sections weighing approximately 30–50 mg were cut from the biopsy center and homogenized in bead tubes (Navy, NextAdvance) with TRIZOL (Ambion). RNeasy spin columns (Qiagen) were used to extract RNA using the manufacturer’s protocol. One microgram of complimentary DNA (cDNA) was reverse transcribed with iScript cDNA Synthesis kits (Biorad). Quantitative PCR was carried out on custom plates on a BioRad CFX384 Touch qPCR analyzer for a panel of 42 genes associated with myogenic, atrophic, adipogenic, fibrotic and inflammatory pathways (Table 1), with cycle threshold determined using a SYBR green fluorophore. On-plate quality assessment was performed to assess gDNA contamination and RNA quality. Each sample was contained on a single plate, negating the need for inter-plate corrections.

**Table 1:** Gene categories, linear regression, and PCA data.

Gene Category	Gene Name (Abbreviation)	p-value	r^2^	1st PC	2nd PC
Muscle structure/myogenesis	Embryonic Myosin Heavy Chain (MYH3)	n.s.	—	**0.194**	0.077
Muscle structure/myogenesis	Myosin Heavy Chain—Type I (MYH1)	6.24E-05	0.41	0.108	**0.211**
Muscle structure/myogenesis	Insulin-like Growth Factor 1 (IGF1)	n.s.	—	0.106	***-0*.*204***
Muscle structure/myogenesis	Cysteine and Glycine Rich Protein 3 / Muscle LIM Protein (CSRP3)	1.77E-07	0.602	0.131	**0.194**
Muscle structure/myogenesis	Ankyrin Repeat And SOCS Box Containing 15 (ASB15)	7.43E-10	0.726	0.144	**0.200**
Muscle structure/myogenesis	Ankyrin Repeat Domain 2-Stretch Responsive Muscle (ANKRD2)	7.88E-09	0.678	0.165	0.163
Muscle structure/myogenesis	Paired box 7 Transcription Factor (PAX7)	8.72E-09	0.676	0.168	**0.185**
Muscle structure/myogenesis	Myogenin/Myogenic Factor 4 (MYOG)	4.93E-08	0.635	0.154	**0.177**
Muscle structure/myogenesis	Myogenic Differentiation 1/Myogenic Factor 3 (MYOD1)	1.08E-07	0.615	0.128	**0.172**
Muscle structure/myogenesis	Myogenic Factor 5 (MYF5)	6.88E-07	0.564	0.160	0.153
Atrophy/Myogenic Inhibition	Myostatin/Growth Differentiation Factor 8 (MSTN)	1.28E-05	0.469	**0.212**	0.108
Atrophy/Myogenic Inhibition	Activin Receptor 2B (ACVR2B)	n.s.	—	**0.217**	0.002
Atrophy/Myogenic Inhibition	Tripartite Motif Containing 63/E3 Ubiquitin Ligase (TRIM63)	n.s.	—	0.169	0.144
Atrophy/Myogenic Inhibition	Forkhead Box O3 (FOXO3)	n.s.	—	**0.233**	*-0*.*068*
Atrophy/Myogenic Inhibition	F-box Protein 32/Atrogin-1/Muscle Atrophy Fbx32 (FBXO32)	3.23E-09	0.697	**0.218**	0.111
Atrophy/Myogenic Inhibition	Caspase-3 (CASP3)	n.s.	—	**0.208**	*-0*.*127*
Atrophy/Myogenic Inhibition	Caspase-1 (CASP1)	n.s.	—	**0.175**	*-0*.*155*
Metabolism	Protein Tyrosine Phosphatase, non-Receptor Type 4 (PTPN4)	n.s.	—	**0.236**	0.018
Metabolism	Mammalian Target of Rapamycin (MTOR)	n.s.	—	**0.227**	*-0*.*055*
Adipogenic	PPARG Coactivator 1 Alpha (PPARGC1A)	1.13E-06	0.549	**0.209**	0.098
Adipogenic	Peroxisome Proliferator-Activated Receptor Gamma (PPARG)	n.s.	—	**0.200**	*-0*.*073*
Adipogenic	Peroxisome Proliferator-Activated Receptor Delta (PPARD)	n.s.	—	**0.223**	*-0*.*071*
Adipogenic	Fatty Acid Binding Protein 4 (Adipcyte-Specific) (FABP4)	n.s.	—	**0.201**	0.007
Adipogenic	CCATT/Enhancer Binding Protein Alpha (CEBPA)	n.s.	—	**0.193**	*-0*.*048*
Adipogenic	Adiponectin (ADIPOQ)	n.s.	—	**0.183**	0.021
Adipogenic	Wnt Family Member 10B (WNT10B)	n.s.	—	*-0*.*060*	0.106
Inflammation	Tumor Necrosis Factor (TNF)	n.s.	—	0.119	*-0*.*038*
Inflammation	Interleukin-6 (IL6)	n.s.	—	0.093	*-0*.*169*
Inflammation	Interleukin-10 (IL10)	n.s.	—	0.047	***-0*.*195***
Inflammation	Interleukin-1 Beta (IL1B)	n.s.	—	0.051	*-0*.*162*
Fibrosis	Platelet-Derived Growth Factor Receptor Alpha (PDGFRA)	n.s.	—	0.140	***-0*.*194***
Fibrosis	Tissue Inhibitor of Metalloproteinase 3 (TIMP3)	n.s.	—	***-0*.*177***	0.128
Fibrosis	Tissue Inhibitor of Metalloproteinase 1 (TIMP1)	n.s.	—	0.093	***-0*.*229***
Fibrosis	Matrix Metalloproteinase 9 (MMP9)	n.s.	—	0.004	*-0*.*154*
Fibrosis	Matrix Metalloproteinase 3 (MMP3)	n.s.	—	*-0*.*026*	*-0*.*148*
Fibrosis	Matrix Metalloproteinase 1 (MMP1)	n.s.	—	*-0*.*023*	*-0*.*135*
Fibrosis	Lysyl Oxidase (LOX)	n.s.	—	0.070	***-0*.*224***
Fibrosis	Fibronectin 1 (FN1)	n.s.	—	*-0*.*016*	***-0*.*219***
Fibrosis	Connective Tissue Growth Factor (CTGF)	n.s.	—	0.113	***-0*.*209***
Fibrosis	Collagen Type III Alpha 1 Chain (COL3A1)	n.s.	—	0.012	***-0*.*228***
Fibrosis	Collagen Type I Alpha 1 Chain (COL1A1)	n.s.	—	0.011	***-0*.*230***
Fibrosis	Transforming Growth Factor Beta 1 (TGFB1)	n.s.	—	0.151	***-0*.*217***

Genes categorized by most relevant category. Coefficient of determination (r^2^) for normalized expression and muscle fraction calculated via histology. Gene weights for the first two principle components are reported. Note that directionality of gene weights indicates genes with opposing expression trends, and does not indicate positive or negative disease effects.

### Gene expression analysis

Raw cycle-threshold values (Ct values) were obtained from all samples and read into a qPCR expression set using the R Bioconductor package HTqPC, and were normalized using the delta-Ct normalization method to obtain gene expression values (*RPS18* and *ACTB* used as reference genes). Note that a maximum Ct of 39 was applied to all genes of interest to allow for statistical comparisons, and that lower values indicate higher expression in this method[15].

To determine the effect of tissue composition on muscle gene expression, linear regression of normalized gene expression and previously measured and reported histological parameters[6] was implemented. The following histological parameters from the previous study[6] were evaluated here: relative tissue fractions of muscle, connective tissue, and fat, along with inflammation (macrophage density) and vasculature (α-SMA+ vessel density and size). Coefficients of determination were calculated for linear relationships between expression values and histological parameters, and were considered significantly predictive when r^2^ >0.2 and Bonferroni-corrected p-values were statistically significant (α = 0.05).

Unsupervised hierarchical clustering using Euclidean distance was then applied to the normalized expression values to determine the ability of gene expression patterns to differentiate muscle-containing samples from those without muscle, and to identify potential sub-clustering of muscle-containing samples. Where appropriate, compositional parameters were compared using a two-tailed t-test (α < 0.05) to determine significant differences in average composition of the sub-group versus the remaining biopsy pool. Additionally, principle component analysis (PCA) was performed on the normalized gene expression values using the R package prcomp[16], in order to better appreciate sample clustering and to identify the genes with the largest effect on variability between samples. Subsequent differential gene expression sub-analyses were performed based on the groups identified by hierarchical clustering and confirmed by PCA.

Differential expression values (delta-delta-Ct)[15] were calculated with the limmaCtData wrapper in HTqPCR for the Bioconductor package limma using a moderated *t-*test[17]. Based on the cluster analysis and histological data available for the RSA biopsies, the intact comparisons described below include only intact biopsies that clustered with muscle-containing RSA samples. Differential expression values were computed for: 1) RSA biopsies with muscle present vs. without muscle, 2) pooled RSA biopsies versus the intact group, and 3) each main RSA cluster compared to the intact group. Differential expression values were also computed between clusters to determine if different sub-groups had significantly different gene expression. In all analyses, muscle content was included as a covariate to correct for the demonstrated effects of muscle content on expression profile, and genes with a Benjamini-Hochberg adjusted p-value < 0.05 were considered significantly differentially expressed. All raw data used in this study may be found in the Supporting Information (S1 File).

## Results

**Fig 2:**
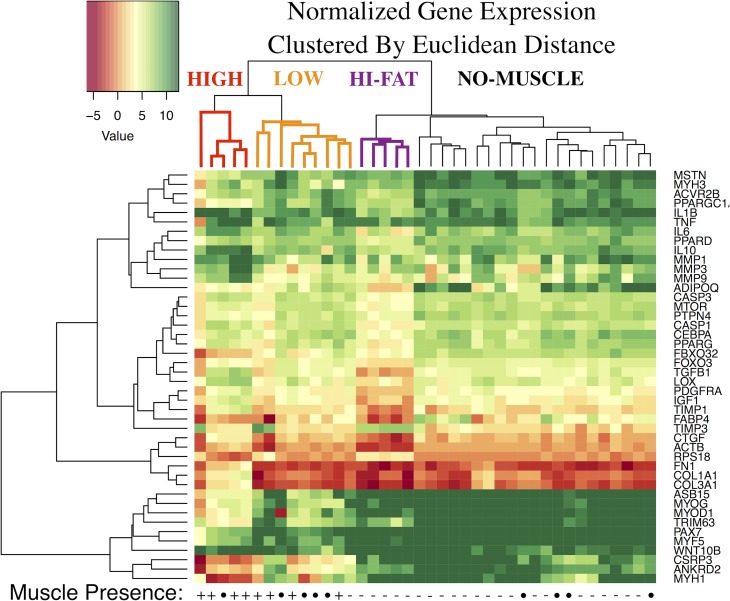
Hierarchical cluster analysis of all muscle biopsies, using Euclidean distance as the similarity metric. The histological presence or absence of muscle is noted on the bottom edge of the heatmap, with INTACT samples indicated by filled circles. Distinct biopsy clusters are denoted by coloring of the dendrogram leaves—the high-expression group (HIGH) is red, low-expression group (LOW) is orange, and high fat group (HI-FAT) is purple.

As discussed in our previous publication[6], only 8/31 (26%) RSA samples contained histological evidence of skeletal muscle (Fig 1B). In samples lacking histologically identifiable muscle (Fig 1C), expression of muscle-specific genes was almost entirely absent (Fig 2, black dendrogram branches). Similarly, muscle content was significantly correlated to the expression of 11 muscle related genes (Table 1); there was no significant correlation between gene expression and any other histologic metric.

Hierarchical cluster analysis of normalized expression values (noting that with the delta-Ct method, greater expression is denoted by negative values) resulted in two primary clusters, with all muscle-containing RSA samples contained in one primary cluster that also included 5/9 intact samples (Fig 2). Within this muscle-containing cluster, samples were further segregated into two groups: a group with higher expression of myogenic, adipogenic, and metabolic genes and lower expression of fibrotic genes which will be referred to as the HIGH cluster (Fig 2, red), and a second group with lower expression of myogenic and atrophic genes and higher expression of fibrotic genes which will be referred to as the LOW cluster (Fig 2, orange). Based on the exclusivity of the main muscle cluster, only intact samples that co-segregated with muscle-containing RSA samples were used for differential expression comparisons between INTACT and RSA sub-clusters.

**Fig 3:**
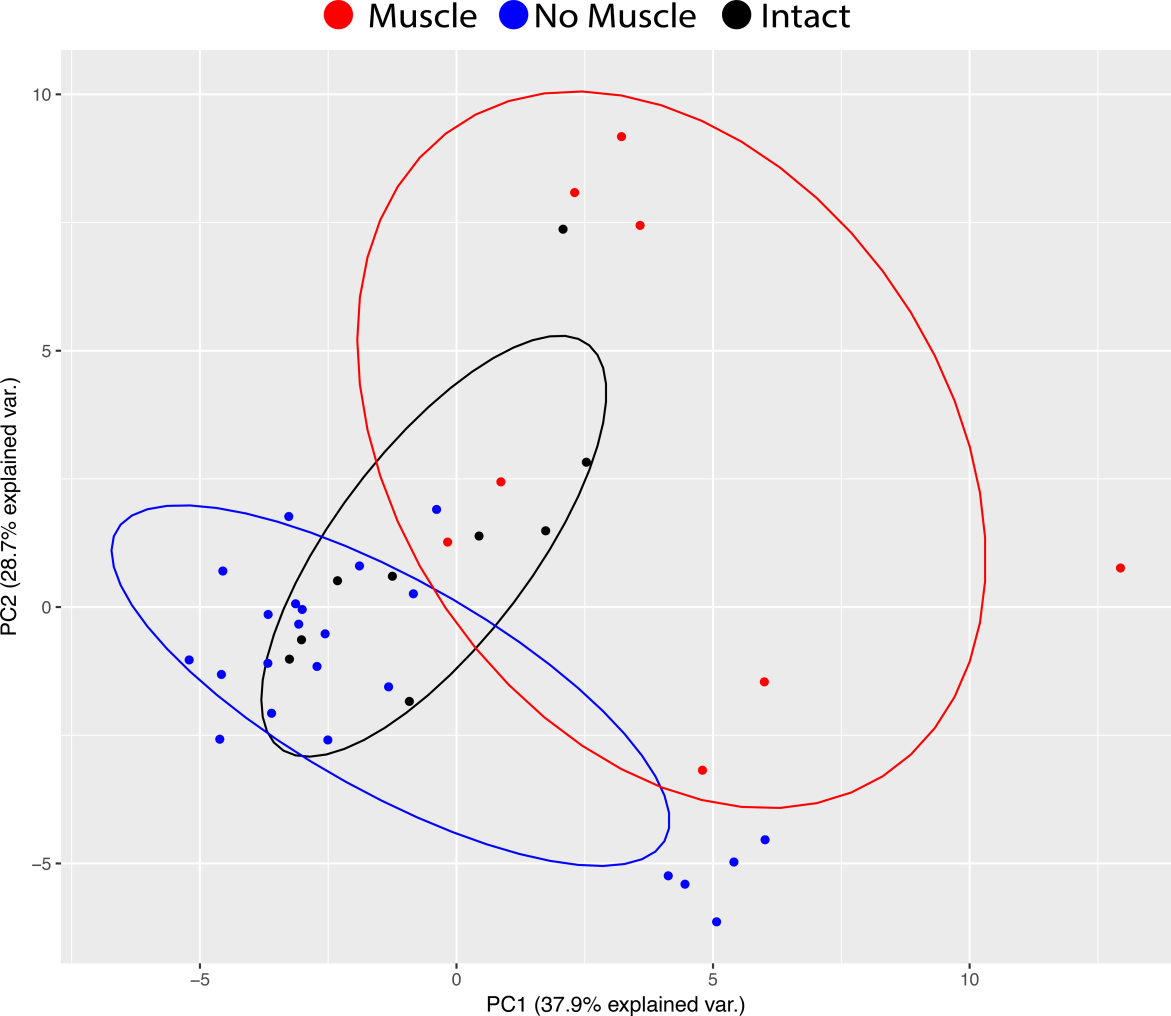
Principal component analysis employed to visualize variability between biopsies. Samples containing histological muscle are red, samples without muscle are blue, and controls are black. Of particular note are the cluster of blue samples in the lower center which correspond to the HI-FAT group in Fig 2, and the high variability in expression among the muscle-containing samples.

The strong effect of muscle presence and relative expression of muscle-related genes was observed in PCA as well. PCA analysis showed a high degree of separation between muscle-containing RSA samples and RSA biopsies without muscle (Fig 3). While the muscle-containing RSA samples tended to be the most variable, RSA samples without muscle formed two clear clusters in the PCA analysis. The smaller cluster (lower center) was found to contain significantly increased fat content via histology when compared to other non-muscle RSA samples (~4 fold, 17.8% vs. 4.3% fat), and will be referred to as the HI-FAT cluster (purple in Fig 2). The remaining samples lacking histological muscle formed a larger cluster defined primarily by expression of fibrotic genes (NO-MUSCLE, black in Fig 2). Similarly, the weighting of the principal components (PCs) reflected these differences across samples, where the first PC (37.9% overall variance) was weighted primarily by myogenic inhibition, apoptotic, and adipogenic genes, while the second PC (28.7% overall variance) was weighted primarily by pro-myogenic and fibrotic genes (Table 1). No clear trend in gene families was observed in subsequent PCs.

**Fig 4:**
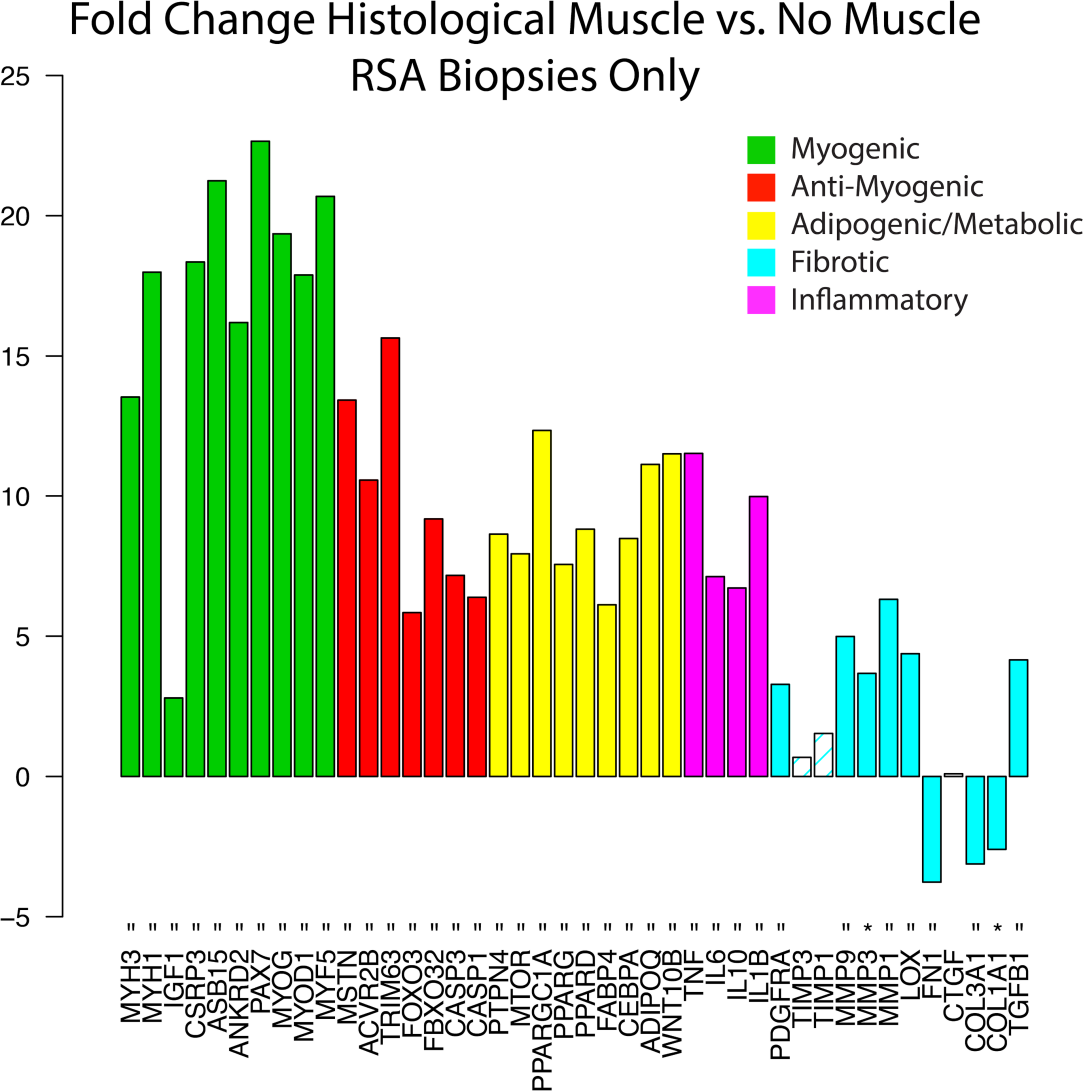
Fold change in expression between the RSA biopsies that contain muscle compared to those without muscle. Solid bars indicate significant up- or down-regulation (p<0.01 and p<0.05 indicated by ‘ = ‘, and ‘ * ‘, respectively). With muscle present, nearly all genes of interest are significantly differentially regulated, with increased expression of muscle- and fat-related genes and decreased expression of fibrosis-related genes.

**Fig 5:**
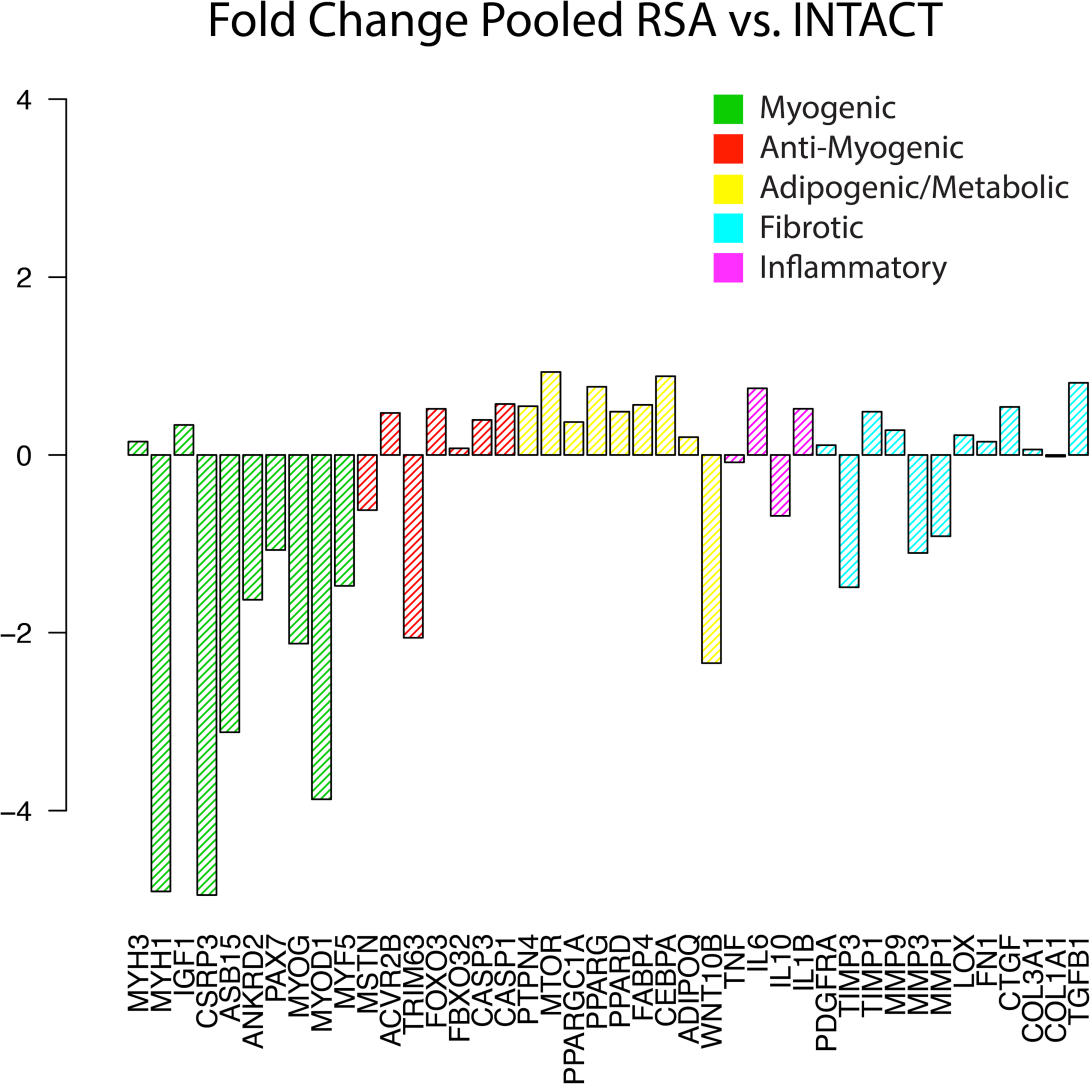
Fold change in expression between pooled RSA biopsies and controls. As a single pool, RSA biopsies are not significantly different from controls, though expression of pro-myogenic genes trended down while atrophic, adipogenic, and fibrotic genes trended up.

Differential expression analysis within the RSA biopsy pool showed that all but three of the analyzed genes (93%) were differentially expressed in samples with muscle compared to those without, with the majority of genes up-regulated in muscle containing samples (Fig 4). In stark contrast, when pooled RSA samples were compared to INTACT, there were no significant differences in expression of any genes, though 8/9 pro-myogenic genes trended toward reduced expression (Fig 5).

**Fig 6:**
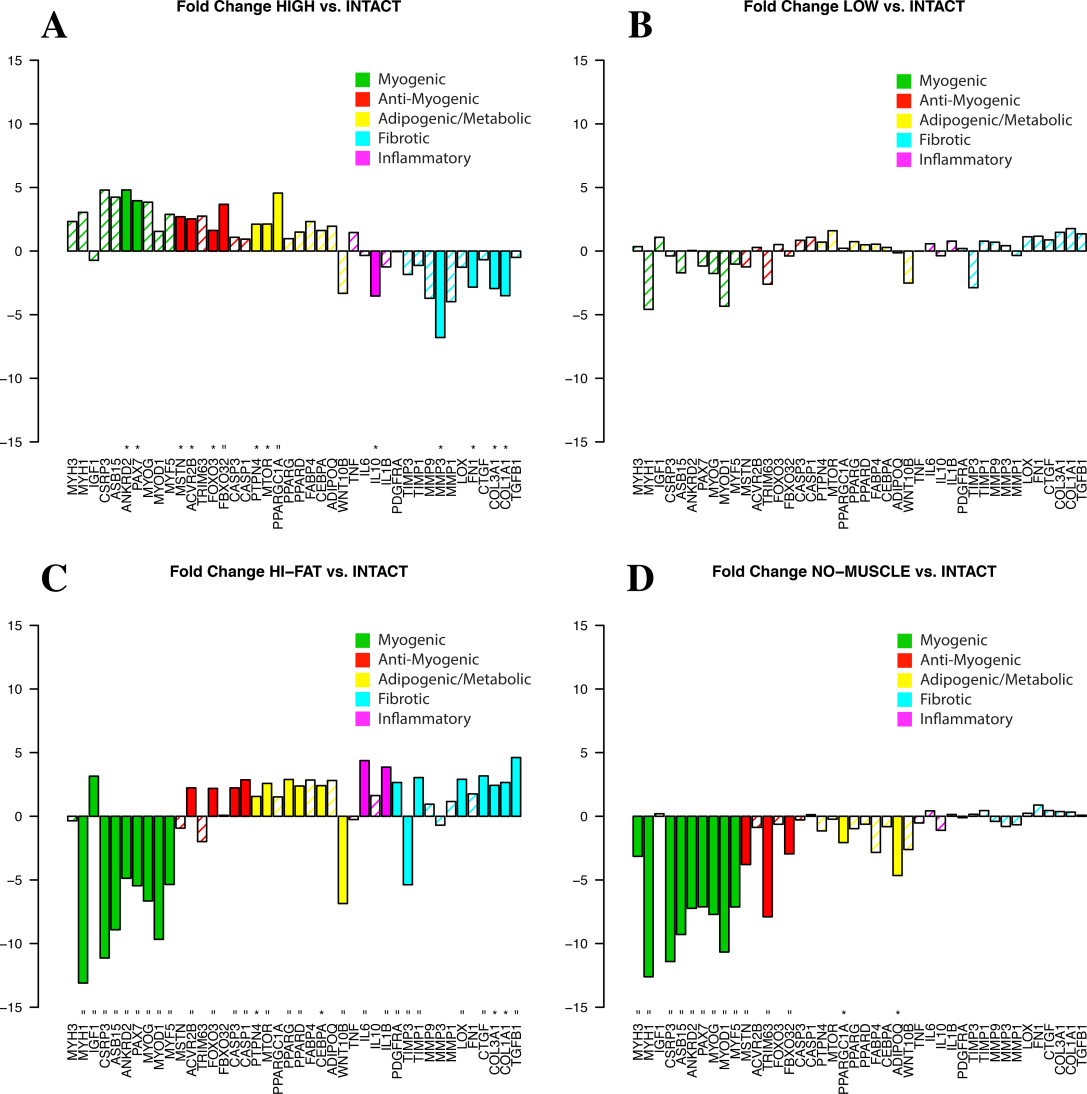
Fold changes in expression relative to INTACT for (A) HIGH muscle group (red in Fig 2), (B) LOW muscle group (orange in Fig 2), (C) HI-FAT group (purple in Fig 2), and (D) NO-MUSCLE group (black in Fig 2). Solid bars indicate significant up- or down-regulation (p<0.01 and p<0.05 indicated by ‘ = ‘, and ‘ * ‘, respectively).

These contrasting results highlight the need for a more detailed segregation and comparison of samples. Compared to INTACT, the HIGH group (red in Fig 2) demonstrated increased expression of both pro- and anti-myogenic genes, with reduced gene expression of three extracellular matrix proteins (Fig 6). However, in the LOW group (orange in Fig 2) no genes were significantly different compared to INTACT. Unsurprisingly, the HI-FAT group demonstrates increased expression of adipogenic genes and decreased muscle-related genes compared to INTACT, while the NO-MUSCLE group shows significant reduction of both pro- and anti-myogenic genes.

**Fig 7:**
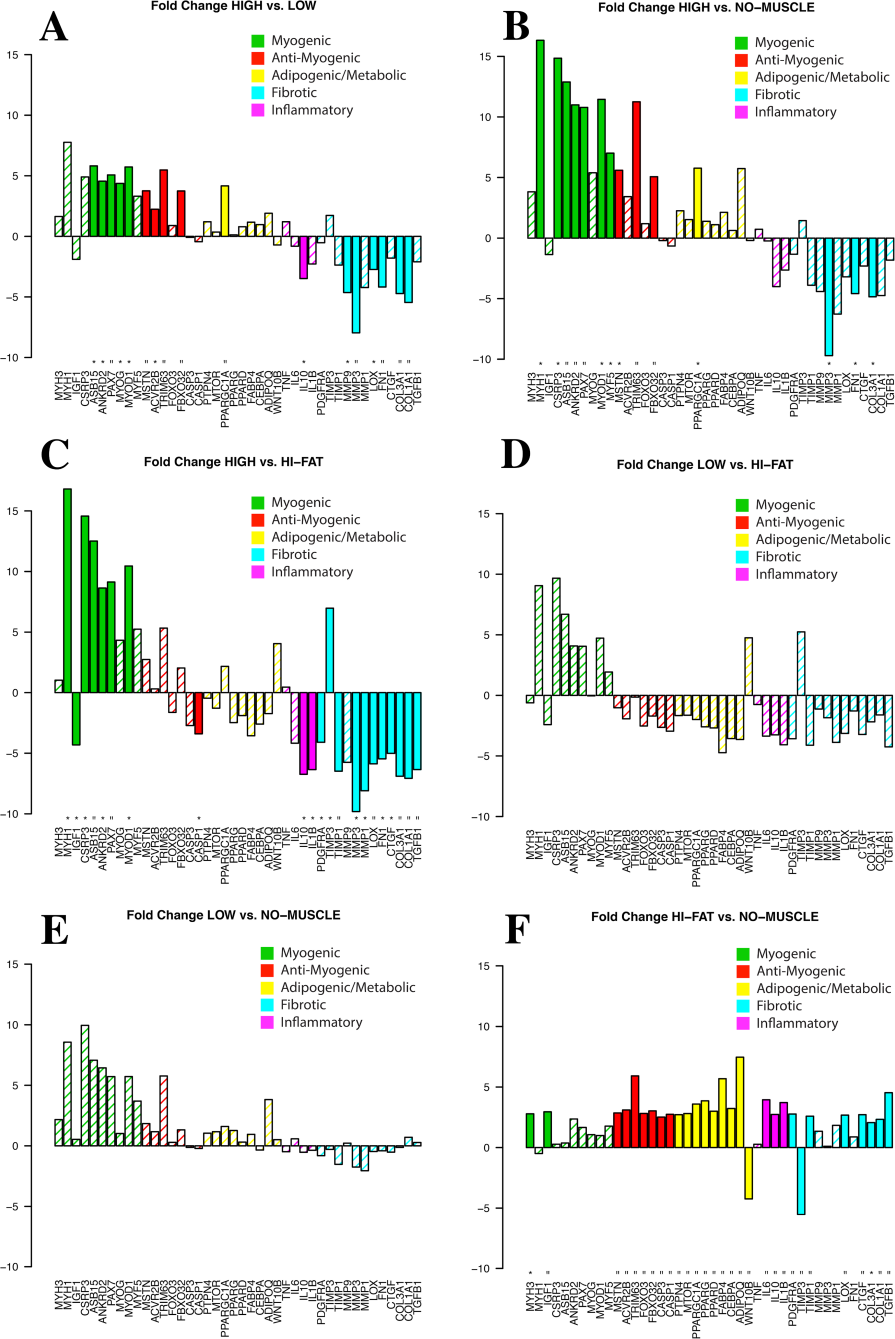
Fold changes in expression between (A) HIGH and LOW expression muscle groups, (B) HIGH and NO-MUSCLE groups, (C) HIGH muscle and HI-FAT groups, (D) LOW muscle and HI-FAT groups, (E) LOW muscle and NO-MUSCLE groups, and (F) HI-FAT and NO-MUSCLE groups. Solid bars indicate significant up- or down-regulation (p<0.01 and p<0.05 indicated by ‘ = ‘, and ‘ * ‘, respectively).

Finally, fold-change expression was calculated between each sub-group (high muscle expression, low muscle expression, high fat content/expression, and the remaining samples without histological muscle or high fat). Compared to LOW group, the HIGH group demonstrated significantly increased expression of both pro- and anti-myogenic genes, with a similar but higher magnitude pattern observed between the HIGH and NO-MUSCLE groups (Fig 7A and 7B). This pattern was also repeated in comparing the HIGH and HI-FAT groups, though additionally the HIGH group showed significantly decreased ECM-related gene expression (Fig 7C). Similar to the analysis relative to INTACT, the LOW group did not show significant differences in expression of any genes compared to either the HI-FAT or NO-MUSCLE groups (Fig 7D and 7E). Compared to the NO-MUSCLE group, the HI-FAT group demonstrated increased expression of both adipogenic and fibrotic genes.

## Discussion

This study highlights the importance of understanding tissue composition, and in particular muscle content, when evaluating gene expression in human muscle biopsies taken from patients with chronic, degenerative disease. Even when screened at the time of surgery and harvested to avoid non-muscle tissue, biopsies are highly heterogeneous and often do not contain any muscle fibers[6]. This poses significant problems in the context of studying how and why muscle is irreversibly lost in RC disease[1] (and diseases with similar chronic and degenerative muscle loss, such as lumbar spine pathology[18] and muscular dystrophy[19–21]). When biopsies are taken from the muscle belly and appear to be muscle both via non-invasive imaging and direct visualization, confirmation of muscle content, let alone broad analysis of biopsy composition, is rarely performed. This data highlights the potential pitfalls of omitting compositional data, where the conclusions reached with and without the context of histology are often drastically different.

The first broad aim of this study, to determine whether and to what extent the composition of a biopsy predicts its measured gene expression, demonstrated mixed results. Perhaps unsurprisingly, linear regression did demonstrate significant predictive capacity of muscle content for 10/17 genes expected to be expressed either solely or primarily by muscle fibers or muscle stem cells. However, the same analysis did not find any significant relationship between fat or fibrous tissue content and any gene family, suggesting that gene expression levels are not necessarily reflective of underlying tissue composition, as is often assumed. At a high level, this suggests that even when accounting for tissue composition, the mechanical and biochemical milieu of the torn RC effect gene expression in multiple cell and tissue types relevant to RC disease progression[22] independent of their relative quantity.

When taken as a single pool, there are no significant differences in the expression profile between RSA and INTACT biopsies. Alone, this finding could either be interpreted as RSA biopsies lacking a distinct gene expression signature compared to ‘healthy’ muscle, or as the INTACT biopsies possessing a ‘pathologic’ expression pattern (as the INTACT samples, while lacking tendon tears, nonetheless came from patients with shoulder pathology). With a more liberal approach, the trends (though insignificant) toward diminished signaling from muscle-related genes and pro-myogenic genes in RSA biopsies could be interpreted as diminished muscle maintenance overall, and indeed would align with previous studies[7, 8]. On the surface this does explain the macroscopic phenomenon; myogenic genes are diminished and muscle is lost over time[23, 24]. But this analysis is based on a highly heterogeneous biopsy pool in which the majority of biopsies *do not contain any muscle*, and therefore would not be expected to express muscle-specific myogenic genes. Indeed, the lack of a significant signal in the pooled RSA analysis appears to be due to high variability across RSA samples, and it is clear that any conclusions reached from this particular analysis, without the context of histology would, at best, be right for the wrong (or at least incomplete) reasons.

A clearer picture begins to emerge when biopsy composition is taken into account. Despite no significant difference in muscle fraction, muscle-containing biopsies segregate into two distinct groups (HIGH and LOW). Compared to INTACT, the HIGH group demonstrated increased expression of both atrophic/anti-myogenic and, to a lesser extent, pro-myogenic genes. This suggests that the HIGH biopsies are still responding to the atrophy pressure of mechanical unloading[25], and are mounting a regenerative response to the muscle fiber damage and degeneration observed histologically[6, 26–28]. Importantly, this interpretation directly refutes the conclusion reached from the pooled analysis, suggesting that continued muscle loss may be attributed to some combination of muscle atrophy and an inability of regenerative processes to match the rate of muscle degeneration, rather than a general reduction in myogenesis.

In contrast, the LOW group did not have any differentially expressed genes compared to INTACT, though myogenic gene expression trended downward. To better understand this finding, we looked to the sub-group comparisons. Surprisingly, the low-expression muscle group did not differentially express any genes compared to either the high fat or no muscle groups, but had increased expression of inflammatory and fibrotic markers compared to the high-expression muscle group. Two competing explanations may account for this finding. One possibility is that this group is more ‘terminally degenerated’, evidenced by its similarity to the high-fat and no-muscle sub-groups. In this case, the similarity of expression to the INTACT group is indicative of pathology—the muscle is mechanically unloaded and contains degenerating fibers, but is not expressing the atrophic genes that are expressed in otherwise healthy unloaded muscle[25, 29, 30], nor is it mounting a normal regenerative response to muscle fiber damage[26, 27, 31–33]. However it is also possible that, based on its similarity to INTACT, the LOW group is in a less advanced stage of disease. Given the difficulty of resolving the time course of disease clinically, a definitive answer to this question from any clinical data set is unlikely; to satisfactorily determine the course of gene expression changes over time will likely require an animal model that adequately represents human disease.

Together, the segregation and interpretation of expression results for the HIGH and LOW groups provide further evidence for the existence of a biological spectrum of RC disease[6], though our understanding of how disease progresses through different stages, how these biological changes relate to clinical scoring systems[4, 34], and the processes that underlie the transition from active to terminal muscle degeneration, remains limited. While strategies have been developed for deconvolution of gene expression data, they rely on accurate understanding of both sample composition and expression in each individual cell population[35]. Even in healthy muscle this strategy would be complicated; fluorescence assisted cell sorting (FACS) could isolate and assay the subset of mononuclear cells expected to be found in the muscle, but there is often debate regarding the relationship between cell surface markers and specific cell populations[22, 36–38] and the isolation process itself may alter expression profiles in the cells of interest. Beyond that, the arguably most important cells, the multi-nucleated muscle fibers, would be lost in this process, as FACS cannot isolate myonuclei. In pathological muscle, where the relative cell fractions for known cell populations change dramatically and there remains the possibility of unexpected and potentially unidentified cell populations, this strategy may miss critical contributors to muscle pathology. Furthermore, though highly prevalent, the number of degenerating fibers in a given biopsy relative to the number of non-degenerating fibers at any single point in time is relatively low. One possible strategy to overcome this limitations is physical isolation of specific regions within the muscle biopsy using laser capture microscopy (LCM), which may allow for more targeted investigation of degenerative mechanisms without the confounding signal of surrounding fat, connective tissue, and non-degenerative muscle fibers. Indeed, LCM and associated downstream analytic techniques to hone in on mechanisms of muscle degeneration are a current focus in our lab.

Despite the fact that histological parameters other than muscle content are not linear predictors of gene expression, which may be explained by the chronicity of disease[23, 39] relative to the phase of active remodeling[40], these data nonetheless demonstrate the importance of interpreting gene expression data in the context of biopsy composition[11, 14]; without definitive evidence for the presence of muscle, there would be no basis for even the broad interpretations presented above. More generally, the disconnect between gene expression and histological findings (where even muscle presence and fat content are only modest predictors of expression levels and sample clustering) further complicates the interpretation of gene expression data not only in this and previous studies[7, 8], but throughout the RC disease literature, where complex interactions of the mechanical and biological environment have a significant impact on several of the tissue types central to RC muscle pathology[22].

A major remaining limitation of this work lies in our inability to histologically characterize our INTACT samples. Because the INTACT patients did have sufficient shoulder symptoms to warrant surgery, it is possible that these samples are not representative of normal, healthy muscle. Supporting this, 44% of INTACT biopsies were excluded from the differential expression analysis based on cluster analysis, though it is important to note that trends in gene expression fold-change were not altered when normalized to the entire pool of INTACT samples. In the same vein, the relatively poor predictive ability of histological parameters outside of muscle content remains a limitation, and points to one of the overarching limitations of studying clinical samples, which is an inherent inability to resolve the time course of disease in the majority of patients[41]. To adequately address these limitations will require either development of a new animal model or validation of an existing model that adequately recapitulates both the atrophic and degenerative phases of human disease[22]. Such a model may then be employed to more accurately define the time course of disease, including the relationship between observed changes in gene expression as they relate to sample composition over time.

## Conclusion

As a group, pooled RSA muscle biopsies provide little insight into gene expression patterns in RC disease, with only trends toward down-regulation of muscle-related genes and up-regulation of a limited number of inflammatory and adipogenic genes. But when muscle content determined via histology is considered, much of the variation in RSA gene expression can be explained—samples containing muscle fibers are unanimously separated from samples without muscle, and further segregate into two categories: one with increased expression of both myogenic and atrophic genes indicative of a responsive muscle undergoing active muscle turnover[42, 43], and another with an expression pattern not significantly different from control samples or samples without muscle, indicating potentially diminished responsiveness to both atrophic and degenerative stimuli. These categories may help explain the variability in rehabilitation found in the patient population; while the first category may represent a patient subset with some potential to respond to intervention[44, 45], the remaining patients (both with and without histological muscle) may represent a terminally degenerated muscle with limited regenerative capacity[1, 46, 47]. Future work in this area should focus on two areas: combining histology-informed interpretations of data with better controlled time series data from clinically relevant animal models, and employing advanced biological tools including FACS and LCM to physically separate and assay specific cell types and degenerative muscle fibers to better characterize the molecular mechanisms that govern progression of muscle loss and fat accumulation. The former strategy will allow for improved understanding of the temporal and spatial relationship between gene expression and tissue composition generally. However, the latter, more targeted approach will allow for a deeper understanding of the processes that govern irreversible muscle loss in chronic musculoskeletal conditions, including a more definitive understanding of the phases that define RC disease. Only by separating the highly heterogeneous mix of cell and tissue types[11, 14] will the cellular and molecular processes that govern RC disease progression from reversible, atrophic muscle loss to terminal muscle degeneration be elucidated. Ultimately, understanding the relationships between gene expression, disease state, and patient outcomes will aid in identifying optimal interventions on a more individualized basis, which will in turn lead to improved patient outcomes.

## Supporting information

S1 File Study data. This file contains all of the raw PCR and histological data for this paper. The ‘Raw Cq Data’ tab contains Cq data, where blank cells represent undetected genes which were set to the maximum Ct of 39 in order to perform statistical analysis. All histology data used for regression can be found in the ‘Histology Data’ tab.
(XLSX)
